# The Effect of Thyroid Hormone, Prostaglandin E2, and Calcium Gluconate on Orthodontic Tooth Movement and Root Resorption in Rats

**Published:** 2015-03

**Authors:** Massoud Seifi, Roya Hamedi, Zohre Khavandegar

**Affiliations:** 1 Dentofacial Deformities Research Center, Shahid Beheshti University of Medical Sciences, Tehran, Iran;; 2 Postgraduate Student of Orthodontics, Dental Research Center, Research Institute of Dental Sciences of Shahid Beheshti University of Medical Sciences, Tehran, Iran;; 3 Dentist, Tehran, Iran;

**Keywords:** Calcium Gluconate, Prostaglandin E2, Thyroid Hormones, Tooth Movement, Root Resorption

## Abstract

**Statement of the Problem:**

A major objective of investigators is to clarify the role of metabolites in achievement of maximum tooth movement with minimal root damage during orthodontic tooth movement (OTM).

**Purpose:**

The aim of this study was to determine the effect of administration of thyroid hormone, prostaglandin E2, and calcium on orthodontic tooth movement and root resorption in rats.

**Materials and Method:**

Sixty four male Wistar rats were randomly divided into 8 groups of eight rats each: 1- 20µg/kg thyroxine was injected in traperitoneally after installation of the orthodontic appliance.  2- 0.1 ml of 1 mg/ml prostaglandin E2 was injected submucosally.  3- 10% (200 mg/kg) calcium gluconate was injected.  4- Prostaglandin E2 was injected submucosally and 10% calcium was injected intraperitoneally.  5- Thyroxine was injected intraperitoneally and prostaglandin E2 was injected submucosally.  6- 20µg/kg thyroxine with calcium was injected. 7- Prostaglandin E2 was injected submucosally with calcium and thyroxine.  8- Distilled water was used in control group. The orthodontic appliances comprised of a NiTi closed coil were posteriorly connected to the right first molar and anteriorly to the upper right incisor. OTM was measured with a feeler gauge. The mid-mesial root of the first molar and the adjacent tissues were histologically evaluated. The Data were analyzed by one-way ANOVA and Student-Newman-Keuls test.

**Results:**

The highest mean OTM was observed in the thyroxine and prostaglandin E2 group (Mean±SD = 0.7375±0.1359 mm) that was significantly different (*p*< 0.05). A significant difference (*p*< 0.05) in root resorption was observed between the prostaglandin E2 (0.0192±0.0198 mm^2^) and the other groups.

**Conclusion:**

It seems that the combination of thyroxine and prostaglandin E2, with a synergistic effect, would decrease the root resorption and increase the rate of orthodontic tooth movement in rats.

## Introduction

Remodeling of the surrounding alveolar bone and cellular changes in the periodontal ligament (PDL) underlie orthodontic tooth movement.[1] Reducing the length of treatment may thus help satisfy patients' demands and even lessen the long term sequel.[2] Increasing mechanical force to reduce the treatment time, a major problem in orthodontic practice, leads to several sequel. A common major complication of orthodontic treatment is apical root resorption.[3-5] Therefore maximum tooth movement with minimal root damage has been a major objective of investigators.[6] One of the factors that are being investigated for their effects on tooth movement is prostaglandins (PGs).[7-9] PGs, especially PGE_2_, are potent multifunctional regulators of bone metabolism.[10] PGE_2_ induces morphologic changes in osteoclasts and osteoblasts via increased intracellular levels of CAMP (cyclic adenosine monophosphate);[11-12] exogenous PGE_2_ increases the mRNA (Messenger RNA) synthesis and protein secretion of the Receptor Activator of Nuclear factor kappa-β Ligand (RANKL).[13] However, there was an increase in the amount of root resorption with increasing the numbers and concentrations of PGE_2_ injections.[14-16]

In addition, various factors influence the amount of root resorption, including the proven effect of systemic calcium.[17] Low levels of calcium causes secondary hyperparathyroidism and induces an increase in the secretion of parathyroid hormone (PTH) as well as vitamin D active metabolites.[18-20] In bone, PTH can induce a rapid release of calcium, but it also mediates long-term changes by acting directly on osteoblasts and indirectly on osteoclasts.[21-22] In osteoblasts, PTH affects cellular metabolic activity, gene transcriptional activity, and multiple protease secretion.[23-25] Its effects on osteoclasts occur through producing RANKL, a protein that plays a crucial role in osteoclast formation and activity.[26] Thus, the increase of bone turnover induced by PTH could accelerate orthodontic movement and root resorption.[27-28] In addition to parathyroid hormone, bone resorption activity is also regulated by l-thyroxine.[29] Thyroid hormone plays a crucial role in normal growth and development of vertebrate bones.[30-31] Administration of high doses of l-thyroxine in rats has been found to increase bone resorption.[32] Thyroid hormones increase osteoclastic bone resorption in rats by stimulating the prostaglandin.[33] Its administration in rats increases the speed of tooth movement.[34] Application of low doses of thyroid hormones may have a protective effect on the root surface and reduce the extent of root resorption.[35-37] The studies performed by Poumpros *et al.*[36] and Shirazi *et al.*[35] showed that thyroid hormone administration in rats not only increased the speed of tooth movement, but also reduced the extent of root resorption.

Goldie and King[19] found that systemic calcium deficiency increased OTM. In the study enrolled by Yamasaki *et al.*,[16, 18] Kohoe *et al.*,[17] and Boekenoogen *et al.*[15] that evaluated the rate of OTM and root resorption after injection of PGE2, revealed that increase in osteoclastic activity resulted in increased rate of OTM and root resorption.

So far, no research has been undertaken on injection of thyroxine with PGE or calcium during orthodontic treatment and its effect on root resorption or tooth movement. 

The aim of the present study is to compare and investigate the synergistic effect of thyroxine with PGE2, and calcium gluconate on orthodontic tooth movement and root resorption in rats. 

## Materials and Method

*Animals*

All animal handling and surgical procedures were approved by the local committee for experimental animal research ethics and conducted according to the Institutional Review Board (IRB) guidelines for the use and care of laboratory animals. This study was approved by the Ethics Committee of the Dental Research Center at Shahid Beheshti University of Medical Sciences.

Sixty four male Wistar rats (6-8 weeks old, 230-300 grams weight) were randomly divided into 8 groups (n=8). They were fed on NIH-36 diet for mice and rats, with a minimum of 1.15 per cent calcium content.

Fresh drinking water was provided every day and they were cared for according to the animal welfare regulations. 

*Appliance design*

**Figure 1:**
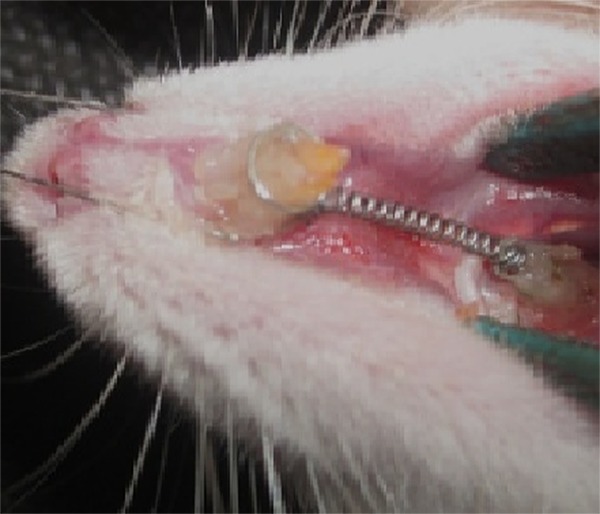
Experimental appliance. An active coiled spring exerted a force of approximately 60 g in the mesial direction

The orthodontic appliance comprised of a 5 mm long NiTi closed coil spring (Orange County; CA, USA) which was connected posteriorly to the right first molar and anteriorly to the upper right incisor by a ligature wire and a force of 60 g was applied (Figure 1). Composite bonding material (3M ESPE, USA) served to fix the ligature wires to the teeth. Measurement of orthodontic tooth movement (OTM), i.e. measuring the distances between the distal of first molar and mesial of second molar, was done by using a feeler gauge (Mitutoyo, Japan) with an accuracy of 0.01 mm.

*Control and Experimental groups*

Both quadrants of the upper jaws of the control (right side under orthodontic force)/normal (no intervention) group of animals were used (eight rats); therefore this group comprised two groups: control and normal. Eight left first molar teeth of these eight animals were not placed under orthodontic force. They represented the normal group and were studied for root resorption only. After insertion of an orthodontic appliance on the right side of the upper jaw, distilled water (0.1 ml) was injected into the mesiobuccal mucosa of the right first molars of the control animals. In this way, the left side of the upper jaw, which was under no force or injection, was considered the normal group and the right side of the upper jaw served as the control. The animals were divided into the following experimental groups:

1. 20µg/kg thyroxine was injected intraperitoneally after installation of the orthodontic appliance in the first group (T group)
2. 0.1 ml of 1 mg/ml PGE2 dissolved in 1% lidocaine was injected submucosally into a similar site for the eight animals in the second group (PGE2 group) with similar orthodontic appliance.
3. 10% Ca (200 mg/kg) was injected intraperitoneally.
4. In the fourth group, PGE2 was injected submucosally and 10% Ca (200 mg/kg) was injected intraperitoneally.
5. In the fifth experimental group, the animals were fit with the same appliance. Thyroxine (20 µg/kg) was injected intraperitoneally and then 0.1 ml of 1 mg/ ml PGE2 (dissolved in 1% lidocaine) was injected submucosally to the mesiobuccal root of the right fist maxillary molars (T+ PGE2).
6. 20µg/kg thyroxine with 10% calcium gluconate was injected intraperitoneally (T+ Ca).
7. In the Seventh group, PGE2 was injected submucosally with 10% Ca (200 mg/kg), and 20µg/kg thyroxine was injected intraperitoneally (T+PGE2+Ca).
8. Distilled water (0.1 ml) was used in the control group.

The injections were administered on the days 0 and 7.

*Histologic evaluation*

The animals were sacrificed after 21 days using vaporized halothane (Parke-Davis; Detroit, MI, USA). The right and left jaw halves of the first eight animals and the right jaw halves of the remnant groups were removed after the experiment period. The specimens were decalcified by formic acid and placed in paraffin blocks. 

The paraffin-embedded samples were cut by a rotary microtome to provide slides. Each sample provided multiple mesiodistal slides of 5 μm thicknesses each. The slides were placed in an oven at 80˚C-110˚C for 30 minutes and were then stained with hematoxylin and eosin (H&E). Adobe Photoshop® software was used to measure the bone histomorphometrical parameters. A grid-sheet used for the preceding evaluation was superimposed in the same way and the numbers of grids were measured and proportion of the pixels in predetermined grid relative to pixels in irregular areas of root resorptions were established for calculating the area of root resorption in mm^2^.

*Statistical analysis*

Descriptive statistics (mean, standard error) of each parameter were calculated for all groups. The scores of tooth movement and root resorption were analyzed by using one-way ANOVA and Student-Newman-Keuls test. The SPSS (version 18) was used and the significance level was set at *p*< 0.05.

## Results

*OTM*

Table 1 illustrates the values obtained regarding OTM in the eight groups with an orthodontic appliance. As the F-test in ANOVA demonstrated a significant difference among the eight groups, a Student's t-test was used to compare the groups in pairs. The mean OTM in the T+PGE_2 _group (mean= 0.7375 mm) was significantly higher than the T, PGE_2_, T+Ca and control groups (*p*< 0.05).

**Table 1:** Mean and standard deviations of orthodontic tooth movement (mm) in the eight experimental groups

**Group**	**Mean**	**Standard** **deviation**	**Range**	**p-value**
T	0.4512	0.1302	0.23-0.69	*p*< 0.05
PGE_2_	0.4700	0.2799	0.21-0.90	*p*< 0.05
Ca	0.2000	0.1010	0.19-0.45	*p*< 0.05
PGE2+ Ca	0.4610	0.0104	0.29-0.57	*p*< 0.05
T+PGE_2_	0.7375	0.1359	0.43-1.05	*p*< 0.05
T+Ca	0.3663	0.04897	0.29-0.45	*p*< 0.05
T+PGE_2_+Ca	0.6525	0.05922	0.55-0.74	*p*< 0.05
Control	0.2313	0.06643	0.14-0.34	N/A

A  *p*< 0.05 was considered to indicate a significant difference

*Root resorption*

**Figure 2:**
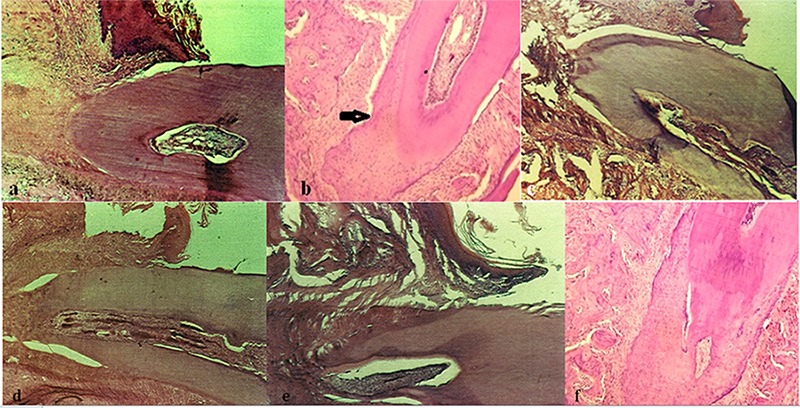
Histological section of the root of a sample in the T (a), PGE_2 _(b), T+PGE_2_ (c), T+Ca (d), T+PGE_2_+Ca (e) and control (f) groups, receiving saline injection and undergoing orthodontic movement, magnification x25. The arrow in PGE_2_ group (B) shows a large resorptive lacuna.

Table 2 illustrates the values obtained for root resorption in the eight groups studied and Figure 2 represents the histological section belonging to the root of a sample from each of the eight groups. Since there was a variance difference in the eight groups, with a *p*-value close to 0.05, and the data did not follow a normal distribution curve, a Kruskal-Wallis test was used to confirm the presence of a significant difference in root resorption among the groups. Multiple range tests were then used to compare groups in pairs, which revealed a significant difference between the PGE2 and T, T+Ca, T+Ca+ PGE2 groups. No significant difference was observed between the other groups regarding root resorption.

**Table 2:** Root resorption (mm^2^) in the experimental groups

**Group**	**Mean**	**Standard** **deviation**	**Range**	**P value**
T	0.001963	0.001342	0.0001-0.00445	0.12
PGE2	0.0192	0.00198	0.20-0.51	0.01*
Ca	0.00202	0.0012	0.0006-0.0046	0.14
PGE2+Ca	0.0031	0.00105	0.002-0.0051	0.09
T+PGE2	0.00418	0.00357	0.0012-0.0125	0.07
T+Ca	0.002194	0.000675	0.0012-0.0035	0.15
T+PGE2+Ca	0.001719	0.001869	0.0011-0.003320	0.21
Control	0.002394	0.001325	0.0006-0.0046	N/A

A  *p*< 0.05 was considered to indicate a significant difference

## Discussion

In this study, OTM occurred significantly faster in T group compared with the control group (*p*< 0.01) which was in agreement with the findings of Shirazi *et al.*[35] The reason for the increase might be the bone-resorptive effect of thyroxine.[30] It directly impacts the bone remodeling,[38] accelerates the osteoclasts activity in rats by stimulating the prostaglandin.[42-44] But thyroid hormones also have an indirect effect via some growth factors that are closely related to bone metabolism, such as insulin-like growth factor I (IGF-I) and IL-1β, produced locally in bone cells by thyroid hormones In the present study, OTM occurred significantly activity.[36-38] Faster in the PGE2 group compared with the control group, which was in line with the findings of Yamasaki *et al.*,[16, 18] Kohoe *et al.*,[17] and Boekenoogen *et al.*[15] This increase might be due to the bone-resorptive effects of PGs after orthodontic loading. Following periodontal injury due to loading, PG is synthesized and PGE2 increases the mRNA synthesis and protein secretion of the receptor activator of nuclear factor kappa-B ligand (RANKL),[13-14] then osteoclastic activity commences, which leads to bone resorption and tooth movement.[16] Thus adding PGE to a live environment may induce bone resorption.[18]

Moreover, OTM occurred slightly lower in the Ca group compared with the control group, which was in accordance with what was found by Goldie and King[19] who observed that systemic calcium deficiency increased OTM. So, the hypoparathyroidism caused by calcium injection in the present study, must have inhibited bone remodeling and resisted the tooth movement.

Combined injection of PGE2 and Ca reduced OTM compared with PGE2 per se; however, despite this decrease it still occurred at a significantly higher rate compared with the control group.

The present results showed that the highest amount of OTM occurred in T+PGE2 group that was significantly higher than T and PGE2 groups. No information was available regarding the combination injection of T+PGE2 during OTM. As mentioned before, thyroid hormones increase osteoclastic bone resorption in rats by stimulating the prostaglandin,[31-39] and exogenous PGE2 increases the mRNA synthesis and protein secretion of the receptor activator of nuclear factor kappa-B ligand (RANKL)[13-14] in osteoblasts. Thus, it can be concluded the bone-resorptive of thyroxine may happen because of RANKL via PGE2.

Combined injection of T and Ca reduced OTM compared with T alone, but despite this decrease it still occurred at a significantly increased rate compared with the control group. Goldie and King[19] found that systemic calcium deficiency increased OTM. Midgett *et al.*[20] demonstrated significantly decreased bone density and increased bone remodeling in animals with hyperparathyroidism, indicating that reduction in bone density would probably facilitate tooth movement within bone.[22] It can be inferred from the above statements that the hypoparathyroidism caused by calcium injection in the present study, must have inhibited bone remodeling and resisted tooth movement,[23-26] whereas this was not the case. This can be explained by the dominant role of T with a dose of 20µg/kg, although a minor insignificant drop was observed in OTM.

Combined injection of T+PGE2+Ca increased OTM, and it was significantly higher compared with the control, T, PGE2, and T+Ca groups. But No statistically significant differences were found between the T+PGE2+Ca and T+PGE2 groups. This can be attributed to the dominant role of T and PGE2. Nonetheless, a minor insignificant drop was observed in OTM due to the hypoparathyroidism caused by calcium injection.[20-25] It was in agreement with the findings of the study conducted by Seifi *et al.*[1] which demonstrated that the tendency towards a reduction of resorption in the PGE2+Ca group may be a result of the transient hypoparathyroidism and diminished resorptive activity subsequent to injection of the calcium compound. The rise in root resorption was significant in the PGE2 group compared with the other groups (Figure 2b).

The present results suggest that the thyroxine-treated animals have shown significantly less root resorptive lesions than the PGE2 group. These data substantiate the findings of the studies by Poumpros *et al.*[36] and Shirazi *et al.*[35] that administration of lower doses of thyroxine reduces force-induced root resorption lesions. Baysal *et al.*[34] suggested that administration of low doses of thyroxine might provide a protective role on the root surface during OTM, and in those patients who present spontaneous root resorption lesions.

No significant differences were found regarding root resorption among the T, Ca, PGE2+Ca, T+PGE2, T+Ca, T+PGE2+Ca, and control groups. As mentioned, PGE2 is effective for promoting orthodontic tooth movement and increase the risk of root resorption. But according to the study by Poumpros *et al*.[36] and Shirazi *et al.*,[35] thyroid hormone administration in rats not only increased the speed of tooth movement, but also reduced the extent of root resorption. Furthermore, the results of the present study showed that the effect of thyroxine in protection of root surfaces is more pronounced than the destructive effect of PGE2 on root surfaces.

The tendency towards the reduction of resorption in Ca, PGE2+Ca, T+Ca, T+PGE2+Ca groups may be a result of the transient hypoparathyroidism and the diminished resorptive activity subsequent to injection of calcium compound,[1, 22] in addition to the root protective effect of thyroxine.[35-39] But since no significant difference was found between the T and T+Ca groups, it can be concluded that addition of Ca to T in order to achieve the minimum root resorption cannot recommended.

## Conclusion

The results of the present study indicate that in order to achieve a decrease in root resorption and an increase in OTM, the combination T+PGE2 is useful and there is a synergism effect with T and PGE2. Using an accurate and appropriate combination of local and systemic factors, it might be possible to reduce treatment duration with fewer complications following orthodontic treatment.

However, before clinical usage, the systemic effects of T+PGE2 on the organs must be evaluated. Correspondingly, further studies are necessary to determine the impact of this combination on the alveolar bone by using TRAP staining protocols or vital staining methods. 

## Notes

**Conflicting Interest: **The authors declare no conflict of interest.
